# Perceived parental bonding and cortisol awakening response in people with eating disorders

**DOI:** 10.1192/j.eurpsy.2024.181

**Published:** 2024-08-27

**Authors:** N. Attianese, M. Battipaglia, S. Donato, R. Ceres, R. Cerra, G. Cascino, P. Monteleone, A. M. Monteleone

**Affiliations:** ^1^University of Campania “L.Vanvitelli”, Naples; ^2^University of Salerno, Salerno, Italy

## Abstract

**Introduction:**

Early life experiences may have an impact on hypothalamic–pituitary–adrenal (HPA) axis functioning in eating disorders (EDs). Parental bonding is defined as the parental contribution of care and control to parent–child relationships. We evaluated whether perceived care and protection of parental bonding in childhood and adolescence were associated with HPA axis functioning in adult patients with EDs. The activity of the HPA axis was assessed by measuring the salivary cortisol awakening response (CAR).

**Objectives:**

We evaluated whether parental care and control in childhood and adolescence were associated with HPA axis functioning in adults with EDs. On the basis of literature data on healthy participants, we hypothesized that people with high levels of parental care would show a reduced CAR compared to people with low levels of parental care.

**Methods:**

We admitted patients according to the following inclusion criteria: (a) female sex, (b) age > 18 years, (c) current diagnosis of AN or BN according to DSM-5 criteria, (d) absence of severe physical disorders, (e) no history of endocrine disorders, psychoactive substance use, schizophrenia or other psychoses, bipolar disorders or head trauma. Participants completed the Italian version of the Parental Bonding Instrument (PBI). To measure the CAR, participants were instructed to collect saliva samples at awakening and 15, 30, and 60 min after awakening.

**Results:**

64 women with EDs participated in the study: 37 with AN and 27 with BN. 28 participants reported low levels of both maternal and paternal care while 12 participants reported high levels of care from both parents; 31 participants reported high levels of both maternal and paternal control, while 12 participants reported low levels of control from both parents. When maternal care was entered as between factor in the analysis, the repeated measures 3-way ANOVA showed a significant time effect (F3, 180 = 2.81, p *<* 0.05) and a significant maternal care X time interaction (F3, 180 = 2.99, p *<* 0.05), while, when paternal care was entered as between factor, the repeated measures 3-way ANOVA did not show significant effects for time and no significant paternal care X time interaction.

**Conclusions:**

Our results show an association of perceived maternal care with the time pattern of CAR in female patients with ED, while perceived parental control was not associated with any CAR feature in EDs. Maternal control, paternal care and paternal control were not associated with any difference in the CAR.

**Disclosure of Interest:**

None Declared

